# The Interplay between PP2A and microRNAs in Leukemia

**DOI:** 10.3389/fonc.2015.00043

**Published:** 2015-02-20

**Authors:** Peter P. Ruvolo

**Affiliations:** ^1^Department of Leukemia, University of Texas MD Anderson Cancer Center, Houston, TX, USA

**Keywords:** PP2A, microRNA, leukemia, signal transduction, AKT

## Abstract

Protein phosphatase 2A (PP2A) is a serine/threonine phosphatase family whose members have been implicated in tumor suppression in many cancer models. In many cancers, loss of PP2A activity has been associated with tumorigenesis and drug resistance. Loss of PP2A results in failure to turn off survival signaling cascades that drive drug resistance such as those regulated by protein kinase B. PP2A is responsible for modulating function and controlling expression of tumor suppressors such as p53 and oncogenes such as BCL2 and MYC. Thus, PP2A has diverse functions regulating cell survival. The importance of microRNAs (miRs) is emerging in cancer biology. A role for miR regulation of PP2A is not well understood; however, recent studies suggest a number of clinically significant miRs such as miR-155 and miR-19 may include PP2A targets. We have recently found that a PP2A B subunit (B55α) can regulate a number of miRs in acute myeloid leukemia cells. The identification of a miR/PP2A axis represents a novel regulatory pathway in cellular homeostasis. The ability of miRs to suppress specific PP2A targets and for PP2A to control such miRs can add an extra level of control in signaling that could be used as a rheostat for many signaling cascades that maintain cellular homeostasis. As such, loss of PP2A or expression of miRs relevant for PP2A function could promote tumorigenesis or at least result in drug resistance. In this review, we will cover the current state of miR regulation of PP2A with a focus on leukemia. We will also briefly discuss what is known of PP2A regulation of miR expression.

## Introduction

The cellular signaling pathways that control cellular homeostasis are complex and involve a diversity of activators and suppressors. As signal transduction is a dynamic process, the elements involved in turning off kinases are just as important as those that activate the signaling cascade. Protein phosphatase 2A (PP2A) is thought of as a global negative regulator of signaling. In reality, PP2A is not a single enzyme but rather a family of protein phosphatases that vary in the substrates they target, in the cell types where they are expressed, in the cellular compartments where they are found, and how they are regulated (1–5). The diversity of function lies in the PP2A structure. The enzyme is a heterotrimer that consists of a catalytic core that is responsible for the dephosphorylation event as well as a regulatory subunit that controls substrate specificity and cellular localization. The catalytic core is comprised of the catalytic C subunit which has two isoforms [PPP2RCA aka Cα and PPP2CB aka Cβ; Ref. (6)] and the scaffold A subunit which also has two isoforms [PPP2R1A aka Aα and PPP2R1B aka Aβ; Ref. (7)]. Each isoform is located on a separate chromosome in humans. Though for both A and C subunits the isoforms are >80% homologous by protein sequence, there are distinct differences between each isoform (6, 7). Thus, there are four different catalytic cores that could influence PP2A activity. The B regulatory subunit determines the substrate specificity and cellular localization of the resulting PP2A isoform. There are at present at least 17 different B subunit proteins that are members of at least three families [i.e., PR55/B; PR61/B′; and PR72/B″; Ref. (1–4)]. PP2A is an obligate heterotrimer so monomeric B subunits are degraded (8, 9). Modification of components of the catalytic core influences binding affinity of the B subunits and thus modulates PP2A function (1). Furthermore, post-translational modification of B subunits can affect sub-cellular localization of the PP2A isoform and influence, which proteins are targeted. We have found the B56α subunit (PPP2R5A) when phosphorylated at serine 28 re-localizes from the nucleus to the mitochondria (10). While nuclear B56α likely supports survival signaling, in the mitochondria the B subunit dephosphorylates and inactivates BCL2 to support pro-death function. Depending on phosphorylation status, the PP2A isoform with B56α can either support or impede survival (10). Thus, cell growth and survival of any given cell depends on it having the appropriate PP2A isoform(s) in place (with the required subunit modifications) to regulate the signaling cascades that are critical for its cell type. In cell that fails to express the PP2A isoforms required for its cell type, it is plausible that the result would be aberrant activation of any number of signal cascades that promotes tumorigenesis or supports drug resistance in the malignant cells.

Suppression of the PP2A family to promote global activation of cellular kinases can be achieved by targeting the catalytic core. Mechanisms include post-translational modification to inactivate protein phosphatase function [e.g., phosphorylation or methylation of the catalytic subunit; Ref. (11–13)], involvement of viral inhibitors like the SV40 small T antigen (14–17), or activation of cellular inhibitors such as SET or CIP2A, the former of which is induced by BCR–ABL in chronic myeloid leukemia [CML; (18–23)]. PP2A can be inactivated by genetic means as well. Mutation of PP2A subunits have been reported with the best characterized so far being those found in the Aα and Aβ isoforms (24, 25). Also, chromosome deletion or translocation containing PP2A subunits have been identified. The 5q deletion can include catalytic Cα subunit and loss of this PP2A subunit has been suggested to be important in myelodysplastic syndrome (MDS) as determined by the List Laboratory (26, 27). The topics of genetic and post-translational control of PP2A and role of SET and CIP2A are covered elsewhere in this Research Topics series. This review will focus on the emerging role of microRNAs (miRs) to regulate PP2A and will also include how at least one PP2A isoform has been shown to regulate miR expression. The interplay between the protein phosphatases and miRs suggest an elaborate feedback mechanism exists to serve as an extra level of control for signal transduction.

### PP2A in role in leukemia

We have some knowledge of PP2A role in CML and Philadelphia chromosome positive acute lymphoblastic leukemia (Ph+ ALL) thanks in large part to the work of the Perrotti group in their studies on BCR–ABL regulation of PP2A in these diseases (18–23). In CML, activation of PP2A is an important part of the mechanism of killing the malignant cells. Furthermore, activation of PP2A by FTY-720 or like drugs has anti-leukemic properties including for acute myeloid leukemia (AML) cells (18–21). Still, little is known about the role of PP2A in leukemia and other hematologic malignancies at present. AML remains a highly fatal disease despite our best efforts to develop novel therapies. Strategies to design tailored therapy have been in vogue but a problem arises that AML patients with the poorest outcome likely have multiple survival kinase cascades activated. Kornblau and colleagues reported that simultaneous activation of protein kinase B (AKT), protein kinase Cα (PKC α), and extracellular signal regulated kinase (ERK) is very detrimental to the AML patient (28). Up to 80% of AML patients have phosphorylated AKT and activation of the kinase is associated with poor prognosis (29–31). Mutations of upstream AKT signaling activators such as Fms-related tyrosine kinase (FLT3), c-KIT, or RAS are found in AML patients (29–31). Targeting mutated enzymes like FLT3 could suppress the aberrant induction of AKT. Though mutation of receptor tyrosine kinases (RTK) like FLT3 has been suggested to be essential for AML, the recent report in *Cell* from Welch and colleagues on genomic screening of mutations in AML indicate that less than half of AML patients have a RTK mutation suggesting that other mechanisms are necessary to activate leukemic signaling pathways (32). Gallay and colleagues determined that reduced PP2A activity was associated with increased phosphorylation of AKT in AML patient samples (33). The Odero Laboratory in Spain has done extensive studies of PP2A and SET in AML (34–36). They determined that elevated SETBP1 (which stabilizes SET and supports suppression of PP2A) was associated with poor survival outcome in AML patients (34). Like Gally et al. (33), they found that PP2A activity was reduced in AML patient samples and suppressed activity of the protein phosphatase likely involved multiple mechanisms including phosphorylation of the C subunit, inhibition by SET or CIP2A, or dysregulation of subunit expression (35). Our own work has determined that suppressed expression of a specific B subunit, B555α, is critical in AML patients resulting in shorter remission duration and increased activation of AKT and PKC α (37, 38). PP2A in acute lymphoid leukemia has not been studied as well in the clinical setting but pre-clinical models suggest that the protein phosphatase family is important in regulation of apoptosis via BCL2 and NOTCH pathways (10, 39–41).

## The Role of miRs in Leukemia

The discovery of miRs was made in *C. elegans* in 1993 when the lin-4 gene product was identified as a RNA product that was complimentary to the lin-14 gene (42). In 2002, George Calin with Carlo Croce discovered that the 13q14 deletions found in chronic lymphoblastic leukemia (CLL) resulted in loss of miR-15 and miR-16, resulting in over expression of anti-apoptotic proteins such as BCL2 (43). An explosion of studies have occurred linking expression of a wide variety of miRs to various cancers [reviewed in Ref. (44–46)]. The role of miRs in cancer has proven to be complex. miRs can either act as tumor suppressors or tumor promoters depending on their targets (44–46). For instance, the let-7 family of miRs tends to act as tumor suppressors by targeting a number of pro-survival molecules such as RAS, BCL-X_L_, and MYC (46–49). Recent work from the Andreeff Laboratory identified a novel mechanism for the chemokine receptor CXCR4 in the regulation of let-7 (50). In leukemia, a number of miRs have been identified that are important in leukemia cell biology and drug resistance (51–58). Some of these miRs, like miR-15, are tumor suppressors that are reduced or lost in cancer cells. miRs play a critical role in cell differentiation. There has been intensive investigation of miR-155 in a variety of leukemias (59–63). The Baltimore group found that overexpression of miR-155 in murine hematopoietic stem cells resulted in altered hematopoiesis skewing toward granulocyte/macrophage population and mice exhibited features of myeloid neoplasia (59). Importantly, the miR was found to target a number of genes that regulate myeloid differentiation including PU.1. In that and subsequent studies, miR-155 was found to be associated with poor survival outcome in AML patients (59, 61, 62). miR-181 family members, on the other hand, support myeloid differentiation by targeting homeobox (HOX) genes such as HOXA9 and other myeloid regulatory genes (64, 65). While still the role of many miRs in leukemia remains unknown, a growing number of these ncRNAs are clearly important in leukemia biology and may be considered for targeting in future therapeutic strategies.

## miR Regulation of PP2A Subunits

The B subunit regulation mechanism best studied is the one involving proteolytic control. Alternative mechanisms could include regulation of PP2A genes by transcription factors or miRs. A comprehensive study of the A α gene was done by Chen and colleagues and identified a number of transcription factors including SP-1 that control expression of the scaffold gene (66). In hepatocellular carcinoma (HCC), a single nucleotide polymorphism (SNP) mutation in the PP2A A α promoter affects regulation by NF κB (67). The study found that individuals from Southern China with the SNP (rs11453459) were less likely to develop HCC as these individuals exhibited greater expression of the PP2A scaffold gene mediated by NF κB (67). Other examples of gene regulation of PP2A subunits include Ikaros suppression of transcription of the C α subunit (68). Unfortunately, little is known of how PP2A B subunit genes are transcribed.

**Figure 1:**
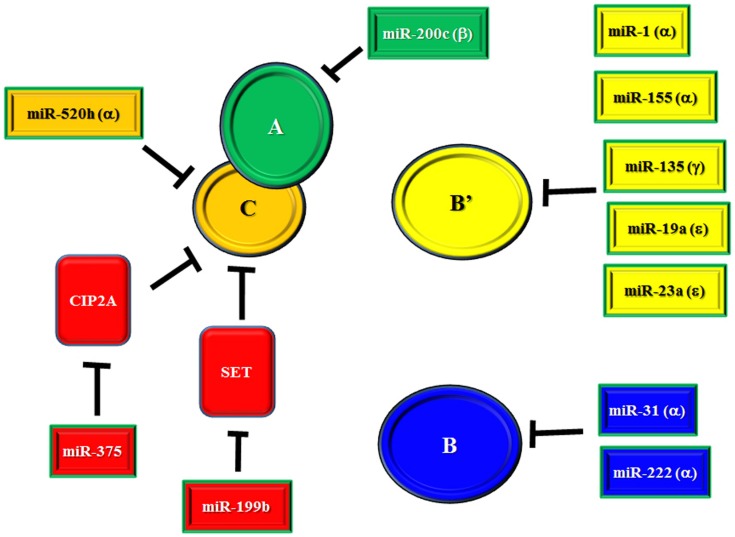
**miR regulation of PP2A and PP2A inhibitors**. Depicted in the figure are identified miRs that target the PP2A catalytic core subunits A and C, various regulatory B subunits, and cellular inhibitors SET and CIP2A.

There is an ongoing effort to characterize the regulation of PP2A subunit gene expression by miRs (69–80). miRs that have been identified as suppressors of PP2A subunit expression are listed in Table 1 and depicted in Figure 1. Also included in Table 1 and Figure 1 are miRs that have been found to target SET and CIP2A as suppression of these PP2A inhibitors would allow for full and potent PP2A activity (81, 82). Regulation of B55α by miR-222 may be important in leukemia as this miR is elevated in MDS patients and the levels of the miR were even higher in patients that progressed to AML (58). Effects on differentiation may be important as many miRs including miR-222 regulate myeloid differentiation (83–86). In addition, miR-222 has been found to be overexpressed in some groups of AML patients and CLL patients with refractory disease (56, 87). NOTCH regulation of miR-19 is critical for T-ALL leukemogenesis and one of the critical targets is B56ε (77). Suppression of B56ε has been shown to be important in AML (36), though the significance of miR-19 in AML has yet to be determined. A lymphoma study revealed that miR-135 targets include B56γ (80). This B subunit is a regulator of p53 and mutations of B56γ have been identified in a number of solid tumor derived cell lines (88). While it is not known if miR-135b is important in leukemia, the miR has been shown to be up-regulated during megakaryocyte differentiation, so the miR plays a role in the biology of at least some hematopoietic cells (85). A greater understanding of the role for miRs in regulating PP2A will be vital to further our understanding of signal transduction control in leukemia and other cancers.

**Table 1:** **List of miRs reported that regulate PP2A subunit or regulators**.

PP2A subunit or regulator	miR identified	Cancer or disease involved	Reference
Cα (PPP2CA)	miR-520h	Breast cancer	(69, 70)
		Cervical cancer	
Aβ (PPP2R1B)	miR-200c	Esophageal cancer	(71)
B55α (PPP2R2A)	miR-222	Hepatocellular carcinoma	(72)
B55α (PPP2R2A)	miR-222	Lung cancer	(73)
B55α (PPP2R2A)	miR-31	Lung cancer	(74)
B56α (PPP2R5A)	miR-1	Heart disease	(75)
B56α (PPP2R5A)	miR-155	Infection (macrophage response to bacteria)	(76)
B56ε (PPP2R5E)	miR-19a	Acute lymphoblastic leukemia	(77, 78)
B56ε (PPP2R5E)	miR-23a	Gastric cancer	(79)
B56γ (PPP2R5C)	miR-135	Lymphoma	(80)
SET	miR-199b	Choriocarcinoma	(81)
CIP2A	miR-375	Oral cancer	(82)

*Listed are reported miRs that target the PP2A catalytic core subunits A and C, various regulatory B subunits, and cellular inhibitors SET and CIP2A. Cancer or disease state associated with each report is listed*.

## Novel Feedback Loops for Signal Transduction: Possible Role for miRs and PP2A

**Figure 2:**
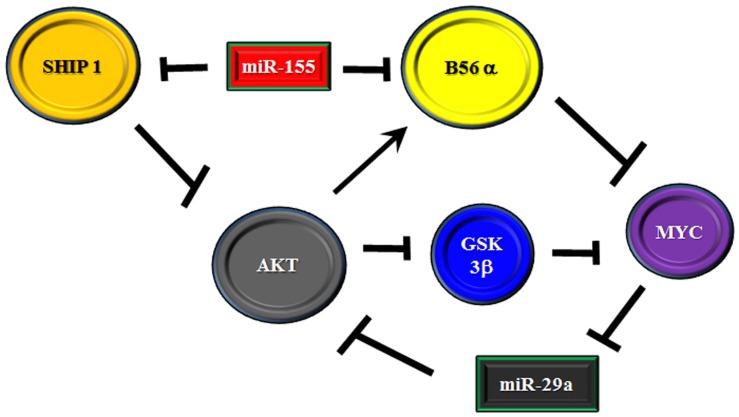
**Model of feedback loop in B56α/AKT/MYC axis involving miRs**. Model is presented where the B56α PP2A subunit and PTEN are negatively regulated by miR-155. The miR may act as a rheostat for AKT signaling as one the one hand PTEN suppression activates AKT but the miR also suppresses the B subunit which is positively regulated by AKT. The B subunit negatively regulates MYC and MYC supports expression of AKT targeting miR-29 so another layer of feedback may exist via MYC/miR-29.

It has been demonstrated that miR-29a is critical for myeloid differentiation and the miR has been shown to be reduced in AML (88, 89). Gong and colleagues recently identified a feedback loop involving the miR-29 family members, AKT2, and MYC (88). The authors found that miR-29 targets AKT which could contribute to the miR’s tumor suppressor activity in AML. Consentient with a role for MYC as a negative regulator of miR-29a, over expression of MYC in AML-derived cell lines resulted in suppression of miR-29a with induction of AKT2 expression while introduction of miR-29a into cells blocked MYC expression and AKT2 levels were reduced. AKT regulation of MYC is likely via suppression of AKT which regulates MYC proteolysis. However, AKT has been shown to support expression of the PP2A B subunit (i.e., B56α) that negatively regulates MYC (10, 90, 91). Thus, an elaborate feedback mechanism to regulate AKT signaling could be mediated by interplay between miR-29a, MYC, and PP2A. To further complicate matters, miR-155 has been identified as an important tumor promoter in AML, high risk MDS, and CLL (61–63, 92–94). One of the targets associated with miR-155 is SHIP 1, a member of the inositol polyphosphate-5-phosphatase family (63, 94). As SHIP 1 is a negative regulator of AKT, suppression of the phosphatase would result in activation of AKT. As miR-155 has been shown to be a prognostic factor for cytogenetic normal AML patients (62), it would be plausible that miR-155 suppression of SHIP 1 could promote signaling in patients that would likely lack activating mutations. Interestingly, miR-155 has now been shown to target B56α (76). Thus, we have another layer of complexity in the putative miR/PP2A regulatory axis to control AKT in leukemia cells. A hypothetical model is depicted in Figure 2. Though quite complex, the possible feedback regulatory mechanism could be necessary for controlling cell fate and dysregulation could ultimately contribute to leukemogenesis and/or drug resistance.

## PP2A, miRs, and Cancer Stem Cells

A study by Yoon and colleagues using human embryonic stem cells (hESC) demonstrated that (a) PP2A activity increases with concomitant increase in A and C subunit expression in differentiating hESC, (b) introduction of catalytic C subunit into hESC or activation of PP2A using C2-ceramide was shown to induce hESC differentiation, (c) hESC self-renewal is maintained in the presence of the PP2A inhibitor okadaic acid, and (d) suppression of PP2A C subunit expression by siRNA-induced stem cell genes such as OCT-4 and Nanog (95). The study found that stabilization of MYC and activation of AKT was important in maintenance of the hESC. Considering the importance of MYC (96) and AKT (30, 31) in leukemic stem cells, it is plausible that PP2A regulation of both these molecules is important in maintenance of leukemia stem cells (LSC). At present, it is unknown if miRs play a role in regulating PP2A activity in normal or cancer stem cells. An interesting possibility emerges that the C α subunit of PP2A and miR-520h could be involved in this process. Su and colleagues recently demonstrated that miR-520h suppression of the PP2A C α subunit was critical for promoting tumor survival and metastasis of breast cancer and cervical cancer cells (69, 70). The adenovirus type 5 E1A protein was found to act as a tumor suppressor by inhibiting miR-520h expression which resulted in activation of PP2A to suppress pro-survival functions of NF κB and TWIST. Interestingly, a recent analysis of miR expression in hESC cell lines indicated that members of the miR-520 family were highly expressed in those cells (97). While it remains to be determined, it is plausible that suppression of PP2A in stem cells observed by Yoon and colleagues could be due to suppression of PP2A by miR-520h (95). As this miR targets a critical component of the catalytic core (i.e., C α) this miR could potentially suppress any number of PP2A isoforms. Another interesting possibility involves miR-200c and the PP2A A β subunit. Recent studies have suggested that miR-200 family members are critical for maintaining stem cells [reviewed in Ref. (97–99)]. These miRs are positively regulated by MYC, OCT-4, and other stem cell transcription factors (98). The miR-200 c family member was found to suppress the PP2A A β subunit in esophageal cancer, resulting in increased chemoresistance and induction of AKT (71). Considering the important role AKT plays in stem cells, the suppression of this PP2A subunit could be important in supporting cancer stem cells. Furthermore, blockade of one of the PP2A A scaffold subunits by a miR could be another means for suppression of PP2A function by a diverse number of PP2A isoforms. Supporting such a concept, recent work from the Perrotti group has demonstrated that activation of PP2A using FTY-720 in CML cells results in eradication of the leukemic stem cells thus the concept of PP2A regulation of stemness likely is pertinent to LSC as well (100). The role of miRs in stem cell differentiation is an active area of research but the field is just emerging. It remains to be determined if and how miRs and PP2A might interact to influence stem cell properties.

## B55α Regulation of miRs in Leukemia

**Figure 3:**
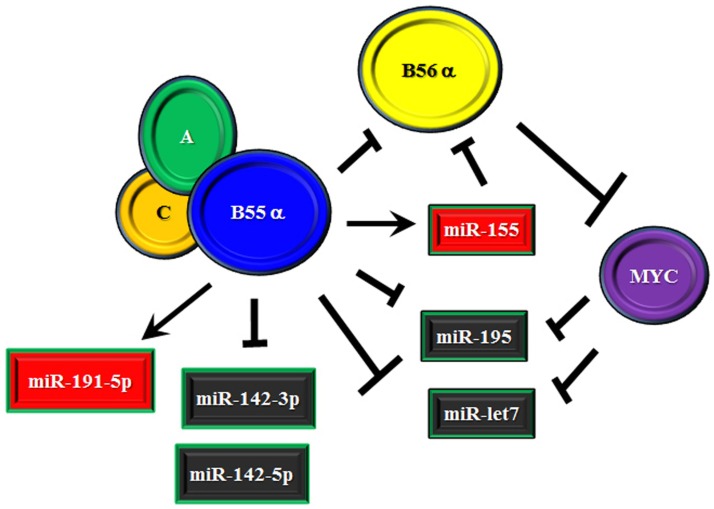
**Model of effect of B55α/B56α competition on miR expression**. Model is presented where the B55α and B56α PP2A subunits compete, resulting in additional regulation mediated by miRs. B55α supports miR-155 which targets B56α so an additional level of suppression exists in the competition between the two B subunits. B56α may have pro-survival function by suppressing B55α mediated expression of miR-191 and/or allowing expression of the miR-142 members, which are inhibited by B55α.

We recently identified a number of miRs that were subject to regulation by the B55α subunit that has been found to suppress AKT and PKC α survival signaling in AML cells (37, 38). The possible role of B55α in regulating miR expression was examined as we had shown the B subunit indirectly supported expression of MYC (38). MYC is known to control a number of miRs (47, 101–103). Suppression of B55α by shRNA in the AML cell line OCI-AML3 resulted in altered expression of a number of miRs. For the most part, reduction of the B subunit led to significant suppression (i.e., >2 fold) of over 30 miRs. This finding would be consistent with possible participation of MYC as MYC has been suggested to have a role in global repression of miRs (47). The miR most affected was miR-1260a, though not much is known about this miR. Among the other miRs that were suppressed are miR-142-3p, miR-142-5p, and miR-195-5p (38). The miR-142 members have been shown to be mutated in lymphoma and AML and thus are of interest to those studying hematologic malignancies (103, 104). Relevant for myeloid leukemia, miR-142-3p has been implicated in control of myeloid differentiation (83). With MYC reduction in the AML cells with reduced B55α, expression of the more prominent MYC targets including those in miR 17–92 cluster were not strongly affected (38). However, induction of miR-195 observed in the cells could be mediated by MYC. MYC has been shown to regulate a number of miRs including miR-195 and it has been suggested that repression of anti-tumor miRs may be critical to MYC’s oncogenic activity (47). While it is not clear the role of miR-195 in leukemia, the miR has been implicated as a tumor suppressor in a number of solid tumor models (105). Targets of miR-195 include cyclins and cyclin dependent kinases (106), MYB (107), and NF κB signaling via IKKα and TAB3 (108). One of the miR-195 targets is the RET tyrosine kinase so perhaps inhibition of this kinase may be important in leukemia (109). Of the miRs elevated in response to suppression of B55α in the AML cells was miR-191-5p and miR-155 (38). The miR-191-5p has been implicated as being detrimental for patient survival in AML (89). As discussed earlier, a critical miR in leukemia and other cancers is miR-155. Interestingly, miR-155 has been shown to target B56α in macrophages (76). This finding and the possibility that B55α supports miR-155 expression suggests a complex regulatory mechanism whereby the B55 subunit can suppress the B56 subunit by competition for the catalytic core and by inducing a miR that targets that competing B subunit (depicted in Figure 3). This regulatory model is currently under investigation in our laboratory.

## Conclusion

The role of miRs as regulators of PP2A and possible control of miR expression by PP2A isoforms is only just emerging. Publications covering miR control of PP2A subunits and regulators of PP2A (e.g., SET and CIP2A) are just emerging. Recent studies suggest that miR control of the PP2A genes is important as results indicate that dysregulation of PP2A targeting miRs can result in induction of signaling pathways associated with the particular PP2A isoform. An example of such a finding involves activation of AKT when miR-222 (which targets the B55α subunit) is elevated (72). The recent finding that miR-155 target genes include a PP2A subunit [i.e., B56α; Ref. (76)] raises the possibility that part of the tumor support properties of this miR may involve suppression of the B subunit. A link between miR-155 and B56α in leukemia cells remains to be established. Surprisingly, the regulation of miRs by PP2A has been less well studied. The well documented relationship between MYC and PP2A would suggest that there would be PP2A regulation of at least MYC sensitive miRs. Interestingly, though B55α serves to positively support MYC expression, there were no effects observed on MYC sensitive miRs in our recent study in AML (38). Still other miRs including miR-142, miR-191, and miR-155 appear to be controlled by the PP2A subunit (38). It remains to be seen how the B subunit regulates these miRs. Are specific transcription factors targeted or are effects mediated by suppression of kinases that phosphorylate transcription factors? Also, a role for other PP2A B subunits in miR regulation is presently unknown. It is likely that other B subunits will exert effects on miR expression and warrant investigation. A better understanding of how PP2A controls miRs and vice versa will provide a better understanding how survival signaling is dysregulated in leukemia and may lead to novel strategies for future therapies.

## Conflict of Interest Statement

The author declares that the research was conducted in the absence of any commercial or financial relationships that could be construed as a potential conflict of interest.
